# Proliferative activity of extracellular HIV-1 Tat protein in human epithelial cells: expression profile of pathogenetically relevant genes

**DOI:** 10.1186/1471-2180-5-20

**Published:** 2005-04-27

**Authors:** Alessia A Bettaccini, Andreina Baj, Roberto S Accolla, Fulvio Basolo, Antonio Q Toniolo

**Affiliations:** 1 Dipartimento di Scienze Cliniche e Biologiche, Università dell' Insubria, Varese, Italy; 2 Dipartimento di Oncologia, Università di Pisa, Pisa, Italy

## Abstract

**Background:**

Tat is being tested as a component of HIV vaccines. Tat activity has been mainly investigated on cells of lymphoid/hematopoietic lineages. HIV-1, however, is known to infect many different cells of both solid organs and mucosal surfaces. The activity of two-exon (aa 1–101) and synthetic (aa 1–86) Tat was studied on mammary and amniotic epithelial cells cultured under low serum conditions.

**Results:**

small concentrations of Tat (100 ng/ml) stimulated cell proliferation. Tat antibodies neutralized the mitogenic Tat activity. Changes of gene expression in Tat-treated cells were evaluated by RT-PCR and gene-array methods. Within 4 hours of treatment, exposure to Tat is followed by up-regulation of some cell cycle-associated genes (transcription factors, cyclin/cdk complexes, genes of apoptotic pathways) and of genes relevant to HIV pathogenesis [chemokine receptors (CXCR4, CCR3), chemotactic cytokines (SDF-1, RANTES, SCYC1, SCYE1), IL6 family cytokines, inflammatory cytokines, factors of the TGF-beta family (TGFb, BMP-1, BMP-2)]. Up-regulation of anti-inflammatory cytokines (IL-10, IL-19, IL-20), a hallmark of other persistent viral infections, was a remarkable feature of Tat-treated epithelial cell lines.

**Conclusion:**

extracellular Tat is mitogenic for mammary and amniotic epithelial cells and stimulates the expression of genes of pathogenetic interest in HIV infection. These effects may favor virus replication and may facilitate the mother-to-child transmission of virus.

## Background

Numerous extracellular roles for the HIV-1 transactivator protein (Tat) have emerged from experiments showing that Tat is taken up by cultured cells, enters the nucleus, and transactivates genes linked to the HIV LTR [1].

Early experiments showing that recombinant Tat was able to inhibit antigen-induced, but not mitogen-induced, proliferation of peripheral blood mononuclear cells indicated Tat as a viral immunosuppressant [2]. This view has been confirmed by subsequent observations indicating that: a) Tat down-regulates HLA class I [3] and class II [4] expression in T-lymphocytes and macrophages, b) Tat represses transcription of the mannose receptor [5] a key molecule in the early response to invading pathogens, c) Tat suppresses CD26-dependent T cell proliferation [6], d) Tat down-regulates TCR/CD3 surface complexes [7], e) Tat induces suppressive levels of alpha interferon in T cells [8], and f) Tat causes human monocytes to secrete interleukin-10 (IL-10) an anti-inflammatory cytokine [9].

Extracellular Tat may stimulate virus replication and disease either through receptor-mediated signal transduction or after internalization and transport to the nucleus [10]. The basic domain of Tat protein (aa 45–56) is required for the binding to the viral transactivation-responsive element (TAR) and for importingextracellular Tat into the cell [11]. Low concentrations of extracellular Tat – similar to those measured in HIV-infected humans, i.e. 2–40 ng/ml [12] – have been shown to promote angiogenesis [13], to stimulate growth and motility of cultured Kaposi sarcoma cells [14], to induce monocyte chemotaxis through the synthesis of platelet-activating factor [15], to stimulate vascular permeability and the recruitment of mononuclear cells [16]. Thus, Tat has been shown to act through several pathways to establish an adequate cellular environment enhancing viral replication.

The multiple pathogenetic activities of extracellular Tat in HIV-infected individuals are unclear, but vaccination with native Tat or detoxified derivatives is being tested to prevent pathogenic events that would otherwise lead to a spreading infection [17,18]. The rationale for this approach is that Tat is conserved among different virus strains [19] and that vaccination might also protect uninfected bystander cells. Human antibodies to Tat correlate with nonprogression of HIV disease [20,21] and have been shown to reduce virus production in HIV-infected cell lines [22]. Remarkably, monkeys immunized with Tat and other HIV antigens were protected from challenge with simian/human immunodeficiency virus [18,23].

For using biologically active Tat as an immunogen, it is desirable to better define its activity on a wide variety of human cells. In this context, it should be considered that most studies have been carried out on cultured cells of lymphoid origin [5,24], whereas HIV-1 is capable of infecting numerous types non-lymphoid cells derived from solid tissues [reviewed in [25]]. As an example, HIV has been shown to affect the viability and functions of lung fibroblasts [26], vascular endothelial cells [27], kidney glomerular mesangial cells [28], as well as epithelial cells derived from the mammary gland [29], intestine [30], and kidney [31].

Our laboratory has shown that primary cultures of mammary epithelial cells (MEC) – that are possibly implicated to the mother-to-child transmission of infection – do express surface CD4, galactosylceramide and CD26 and can be infected by HIV-1 [29]. Infection was associated with the up-regulation of surface HLA class-II molecules and CD26, while the expression of CD4, tissue-specific markers, adhesion molecules and growth factor receptors was downregulated by the virus. Cytopathic effect ensued. Hormones (T3, alpha-estradiol and prolactin) enhanced HIV replication, possibly by stimulating cell proliferation.

Here we analyzed the effects of exogenous Tat protein on epithelial cells of mammary and amniotic origin. It was found that the HIV-1 transactivator induces the above cells to proliferate and that – a few hours after treatment – expression of surface receptors, cytokines and growth factors of pathogenetic relevance in HIV infection is up-regulated.

## Results

### Cell proliferation in response to exogenous Tat

**Figure 1:**
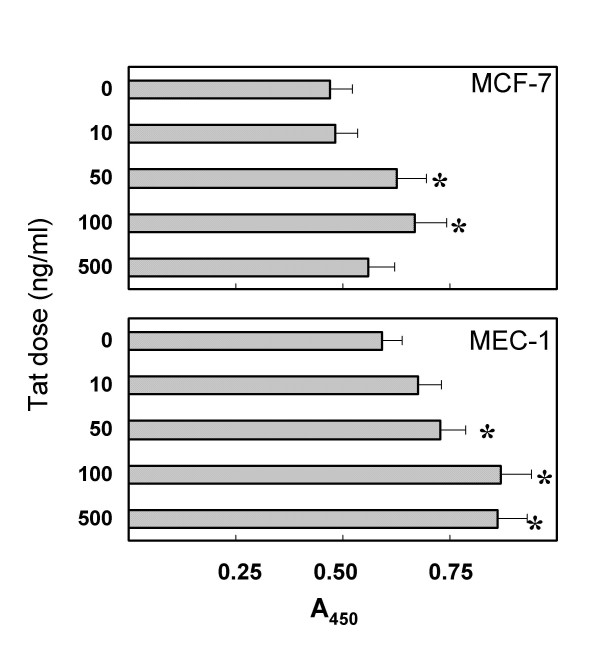
**Growth of mammary epithelial cells exposed to different doses of Tat_101aa_. **Cells were cultured in low-serum medium. Growth was measured by the XTT assay after 48 h of Tat treatment. Each bar represents the average of 3 different tests + SD. *, P < 0.05, as compared with untreated cell cultures.

**Figure 2:**
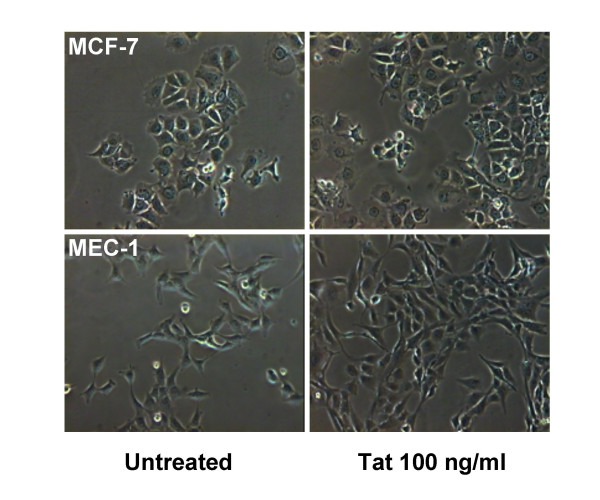
**Monolayers of MCF-7 and MEC-1 cells cultured in low-serum medium and treated or not with extracellular Tat_101aa _(100 ng/ml for 36 hours). **Increased numbers of cells can be observed in Tat-treated cultures as compared to untreated control cultures. Phase contrast; microscopic fields taken with a 10× objective.

**Figure 3:**
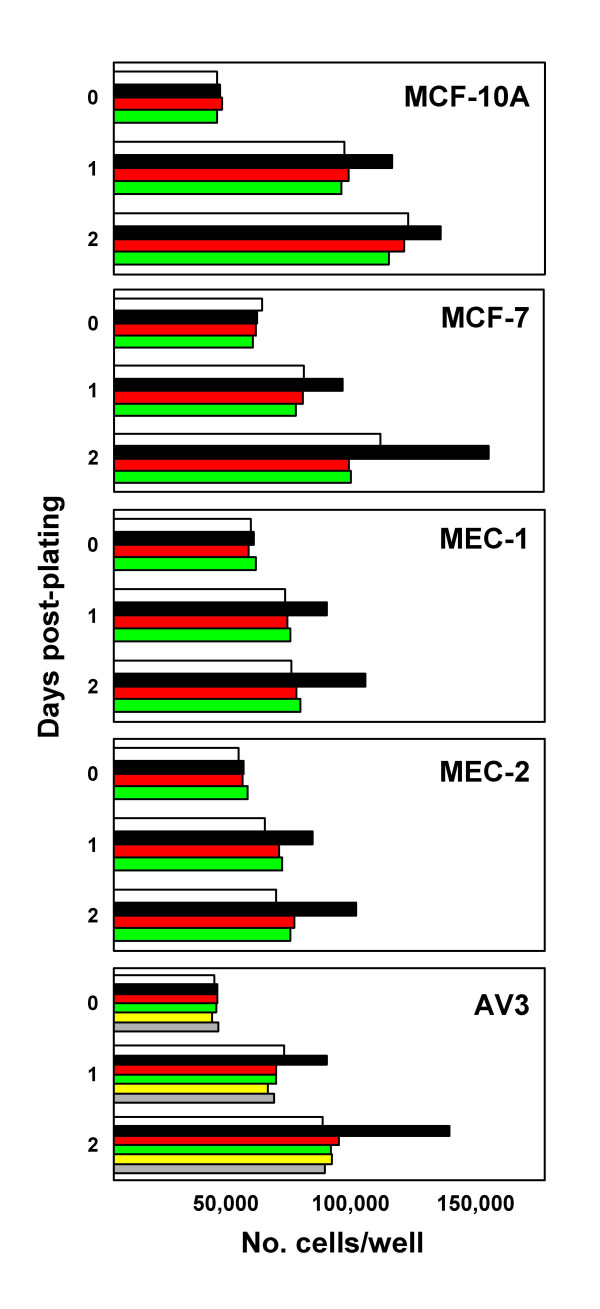
**Neutralization by anti-Tat antibodies of the proliferative response to Tat of five different human epithelial cell lines cultured in low-serum medium. **Cells were either left untreated (open bars) or exposed to 100 ng/ml Tat_86aa _(closed bars) at time 0. Cells were counted by microscopy at day-0, -1 and -2 after plating. Antibody tMAb-B (1 μg/ml) was mixed with Tat before treatment (red bars) or given alone (green bars). Bars represent the mean of three wells. Experiments on AV-3 cells (bottom panel) have also demonstrated the Tat-neutralizing activity of a rabbit polyclonal anti-Tat antibody: anti-Tat plus Tat (yellow bars) and anti-Tat alone (gray bars). Results are expressed as number of cells/well. Each bar represents the average of 3 different tests. Standard deviations of the mean are not reported, but were within 12% of the mean.

Preliminary experiments with a variety of epithelial cell lines have shown that only marginal proliferative responses to Tat were observed when cells were grown in complete medium (i.e., medium containing 10% FBS). In contrast, experiments performed using LSM revealed a marked proliferative effect of Tat on epithelial cells. The activity of various doses of Tat was evaluated with two methods: 1) the XTT assay and 2) cell counts. As shown in Figure 1, the XTT assay showed significant proliferative responses when MCF-7 and MEC-1 cell lines were exposed for 2 days to doses of Tat ≥ 50 ng/ml. Equivalent results have been obtained with recombinant Tat_101aa _and synthetic Tat_86aa _protein preparations (as detailed in the Materials and Methods section). Thus, in subsequent experiments cell cultures were exposed to 100 ng/ml of Tat for different periods of time. In addition to the XTT method, cell proliferation has been measured by direct microscopic counts of cell monolayers. An example of the results is given in Figure 2. Different numbers of adherent cells can be observed in cultures of MCF-7 and MEC-1 cells either untreated or treated with Tat 100 ng/ml for 36 h. Comparable results have been obtained with several different epithelial cell lines. As shown in Figure 3, Tat consistently induced the proliferation of four different lines of mammary epithelial cells and of the amniotic AV-3 cell line. In these experiments it was also shown that a monoclonal anti-Tat antibody was capable of neutralizing Tat proliferative activity. Using the AV-3 cell line, the Tat-neutralizing activity of a rabbit polyclonal antibody was also demonstrated (bottom panel). Unrelated monoclonal and polyclonal antibodies to coxsackievirus B4 failed to neutralize Tat activity (data not shown). The hormone-dependent spontaneously immortalized MCF-10A line gave the lowest response to Tat, whereas consistently higher responses were observed with MCF-7 mammary cells and AV-3 amniotic cells. Thus, these two cell lines have been investigated in more detail to study the influence of exogenous Tat on gene expression profile.

### RT-PCR analysis: Tat modulation of HIV-1 receptors, growth factors, and cytokine transcripts

**Figure 4:**
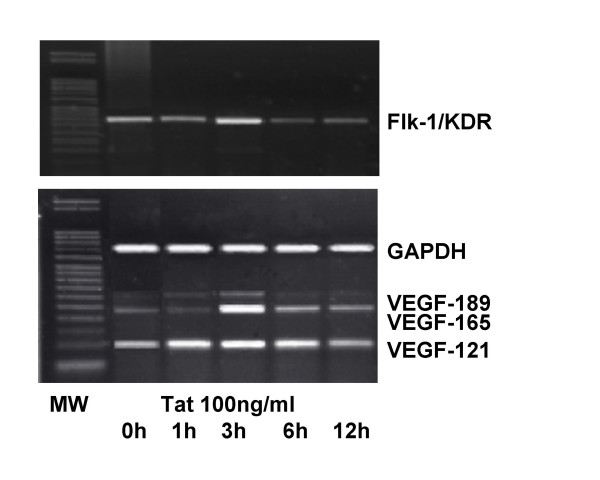
**Semiquantitative RT-PCR of VEGF family genes in MCF-7 cells cultured in low-serum medium and exposed for different times to extracellular Tat_86aa_. **Increased expression of the VEGF receptor-2 (Flk-1/KDR) and VEGF isoform 165. To a lower extent, the VEGF isoform-121 was also up-regulated. The GAPDH signal was used as a control. MW, DNA molecular weight ladder.

Semiquantitative RT-PCR was used to evaluate transcript expression in cell cultures exposed to Tat. Primers for cytokines and VEGF were used in conjunction with a series of published primers (Table 1) to detect alterations of receptor, growth factor and cytokine transcript expression. Different time points were used (i.e., 1, 3, 6 and 12 h post-treatment). As shown by transcript analysis of selected HIV-1 receptors/co-receptors, gene up-regulation was observed early after Tat treatment (i.e., 3–6 h). Results relative to the MCF-7 and MEC-1 cell lines are reported in Table 2. Comparable results have been obtained with other mammary cell lines (data not shown). CD4 is expressed at low-levels in all investigated cell lines. Tat failed to influence CD4 expression. CXCR4, CCR3, and CCR4 transcripts were consistently up-regulated after 3–6 h treatment with Tat. In contrast, CCR1, CCR2, and CCR5 transcripts were not expressed. As shown in Figure 4, up-regulation of VEFG (mainly the 165 isoform) was observed in MCF-7 cells early after Tat exposure (1–3 h). This was concomitant with the up-regulation of VEGF receptor-2 (Flk-1/KDR). This result indicates the possibility of an autocrine loop contributing to Tat-stimulated cell proliferation. VEGF up-regulation was also observed in the other epithelial cell lines using the gene-array technique. Up-regulation of IL-6 and IL-6 receptor has also been detected in MCF-7 and AV-3 cells (data not shown). IL-6 has been shown to contribute to epithelial cell differentiation in both thyroid and the mammary gland [32]. Tat did not influence the expression of IL-8, a neutrophil-attracting cytokine produced by mammary epithelial cells [32]. Exposure to Tat for 4 h was followed by up-regulation of TGF-beta1 and TGF-beta2 in MCF-7 and AV-3 cells. Real time RT-PCR confirmed that the expression of IL-6, IL-6-R, TGF-beta1, and VEGF (isoform 165) was enhanced from 2.4- to 6.5-fold in cells treated with Tat for 4 h. As seen by RT-PCR, expression of CPSF3 [33] was not modified by 1 to 12 h treatment with Tat (data not shown).

### Gene array studies. Genes up-regulated by Tat in both MCF-7 and AV-3 cell lines

Gene-arrays were used to compare gene expression in control untreated and Tat-treated cell cultures. Tables 3 and 4 summarize the results of cultures of MCF-7 and AV-3 cells exposed to Tat for 4 h. Genes that were up-regulated ≥1.5-fold in both cell lines are reported. Up-regulation is expressed as change (n-fold) for each cell line. The last column shows the average up-regulation in the two cell lines. Fifty of 96 genes (52%) related to the cell cycle were up-regulated from 1.7- to 4.6-fold. Twenty-four of 167 genes (14.4%) coding for growth factors, cytokines and their receptors were up-regulated from 1.6- to 3.7-fold.

The up-regulation of a wide spectrum of genes involved in cell cycle regulation that is presented here for two epithelial cell lines confirms the observed proliferative effect of Tat on non-lymphoid cell lines. As shown in Table 3, Tat treatment was associated with increased expression of: a) transcription factors (E2F, E2F-6, MPP2); b) cyclins (cyclin E1, E2, F, B, B2, C, D1, G, H); c) cyclin-dependent kinases and associated proteins (cdk6, cdk7, cdk8, protein regulating cytokinesis-1, cks1p9, cks2); d) cell division cycle proteins cdc27, cdc34, cdc37, cdc45); e) genes involved in DNA repair (MRE11A, nibrin, Hus1, RAD17, RAD50); f) cullins and associated proteins (cul-1, RBX1, Skp1, Skp2, Cul-2, Cul-3, Cul-4A, Cul-4B); g) nuclear factors associated with DNA replication (Ki67, PCNA, rpa, MCM2, MCM4, MCM5, MCM7); h) factors involved in cell cycle arrest (c-abl, chk1, cyclin G2, p18,, P21Cip1, p27Kip1); i) apoptosis-related genes (mdm2, bax).

Tat treatment was also associated with the increased transcription of selected growth factors, cytokines and receptors. Table 4 shows that the following genes were up-regulated in both MCF-7 and AV-3 cells: a) cytokines of the IL-6 superfamily and a signal-transducing molecule (IL-6, IL-11, GP130); b) inflammatory cytokines (LT-b, LTbR, TNFR1, IL-18, MIF); c) chemokines and their receptors (SDF-1, CXCR4, RANTES, CCR3, SCYC1, SCYE1); d) members of the PDGF/VEGF growth factor family (PDGFa, VEGF, VEGF-B, VEGF-C); e) factors belonging to the TGF-beta family [(TGFb1, bone morphogenetic proteins-1 and -2 (BMP1, BMP2)]; f) anti-inflammatory cytokines of the IL-10 family (IL-10, IL-19, IL-20). Exposure to Tat of the AV-3 and MCF-7 cell lines was not associated with downregulation of any of the tested genes.

## Discussion

Our experiments confirm that low concentrations of extracellular Tat are capable of affecting cell proliferation and cellular physiology. We have shown that different epithelial cell lines cultured under low-serum conditions are induced to proliferate by doses of extracellular Tat of at least 50 ng/ml. The Tat dose used in these experiments (100 ng/ml) is close to that measured in the plasma of some HIV-1-infected patients [2–40 ng/ml; [12]]. The values reported [12] could be underestimated because local concentrations of Tat in infected tissues are probably higher as Tat may be sequestered by anti-Tat antibodies and by glycosaminoglycans. Tat produced in HIV-infected cells is released extracellularly and is able to stimulate the growth of Kaposi sarcoma cells at concentrations <1 ng/ml [34]. In the quoted paper, activation of cells by extracellular Tat required concentrations of 100 ng/ml or more. Extracellular Tat has also been shown to enhance the proliferation of germinal center B cells [35] and renal podocytes [36]. Tat is also involved in the progression of tumors arising in Tat-transgenic mice [37]. In lymphoid and neural cells Tat has also been shown to inhibit cell growth and cause apoptosis [38]. Pathways leading to cell death depend on the cell lineage and involve activation of death receptor pathways (i.e., TNF-alpha, Fas, TRAIL), chemokine receptor signaling, cytokine dysregulation, caspase activation, calcium mobilization, and loss of mitochondrial membrane potential.

In HIV replication cycle, Tat is known to interact with the transcription factor IIF and thereby to increase the activity of cdk7, thus allowing RNA polymerase II to transcribe the Tat activation region of the virus LTR [1]. Up-regulation of cdk7 by exogenous Tat has been demonstrated also in this study. Using Tat-transfected cell lines [33], Tat was shown to stimulate mRNA processing through up-regulation of the cleavage and polyadenylation specificity factor 3 (CPSF3). Up-regulation of CPSF3 was held responsible for stimulating viral and cellular gene expression. In contrast, in our experimental model and within 1 to 12 h exposure, Tat failed to induce CPSF3 up-regulation. Cell conditions different from those employed by us may be operative in Tat-expressing cell lines [33].

Increased expression of several transcription factors and cyclin/cdk complexes has been observed in Tat-treated epithelial cells. Genes involved in different phases of the cell cycle are activated, including genes (e.g., bax) linked to cell cycle arrest and negative modulation or apoptosis. These seemingly opposite effects of Tat on cell cycle regulators require some additional comments. Tat may either act directly on the transcription of negative regulators of cell cycle, or their up-regulation may be part of a negative feed back loop generated by an excessive increase of cell cycle inducers. Within this frame, it is of interest to note that extracellular Tat can inhibit p53 functions, thus providing a candidate mechanism through which HIV-1 might contribute to cell cycle dysregulation and malignant transformation [39]. Indeed, though the expression of p53 was not increased in epithelial cells exposed to Tat, the expression of mdm2 (a protein that binds to p53 and represses the p53-induced transcription activation) was strongly up-regulated. This is in contrast to recent findings showing repression of the *mdm2 *gene promoter in cells transfected with Tat constructs [33]. p21Cip1 – an important factor whose expression is regulated by p53 and that blocks cell replication in G1 by inhibiting CDK-cyclin complexes – was strongly up-regulated in Tat-treated cells. Thus, further studies are needed to assess the balance between activation and inhibition of cell cycle genes produced by Tat in cells of different lineages. These studies might help designing innovative antiviral therapies [40]. Measurements of epithelial cell growth in response to extracellular Tat could represent auseful model in this context.

As shown in Kaposi sarcoma cells [13,41], our studies confirm that extracellular Tat up-regulates the expression of VEGF receptor-2 (Flk-1/KDR). Tat also enhanced the expression of multiple angiogenic stimuli (PDGFa, VEGF-165, VEGF-B, and VEGF-C). This was seen both by RT-PCR analysis and gene arrays. In addition to stimulating angiogenesis, these factors are also capable of stimulating epithelial cell growth by autocrine mechanisms. In renal mesangial cells, persistent HIV replication was shown to induce expression of PDGFa/b and TGFb [28], cytokines that mediate mesangial cell proliferation and extracellular matrix deposition.

RT-PCR and gene array studies revealed the increased expression of some chemokine receptors serving as HIV coreceptors (CXCR4, CCR3, and to a minor extent CCR4). CXCR4 is the high affinity receptor for SDF-1, while CCR3 binds RANTES. The enhanced expression of CXCR4 and CCR3 in epithelial cells exposed to Tat suggests that their susceptibility to T- and M-tropic viruses may be increased since these cells express basal levels of CD4 and are permissive to HIV-1 [29]. Enhanced expression of HIV co-receptors has also been shown in monocytes [42], basophils [43], and erythroid cells [44]. Expression of SDF-1, RANTES and other chemotactic cytokines (SCYC1 and SCYE1) was also increased. The ability of Tat to signalthrough chemokines and their receptors is supposed to attract lymphoid cells toward virus producing cells, thus favoring the spread ofinfection. Up-regulation of the IL6 family factors (IL6, IL11, GP130) may promote cell growth [45]. Increased expression of the inflammatory cytokine LT-b (member of the TNF family) may co-operate with IL6 in the reactivation of HIV in CD4 T lymphocytes harboring a latent provirus [46]. Up-regulation of IL18 is also of interest since this cytokine stimulates virus replication. Increased IL18 levels have been reported in HIV-infected patients [47]. Up-regulation of MIF expression may contribute to recruit macrophages at sites of inflammation.

The significance of the increased expression of BMPs is unclear. BMPs have been identified as factors that induce ectopic bone and cartilage production. BMPs constitute the largest subfamily of the TGFb superfamily of growth factors and exert pleiotropic biological effects ranging from regulation of early developmental processes to organogenesis and differentiation of bone, cartilage, epidermal and neural tissues [48]. The increased expression of TGFb found in Tat-treated epithelial cells is a common feature of HIV-1-infected cells and is thought to be an important determinant of immunosuppression [49]. Expression of interferon-alpha (a putative immunosuppressive factor) could not be detected in epithelial cells [8].

Expression of the anti-inflammatory cytokines IL-10, IL-19, and IL-20 appears a remarkable feature of epithelial cell lines. In particular, high-level expression of IL-10 has been detected not only in MCF-7 and AV-3 cells, but also in other mammary and thyroid cell lines. IL-10 is a TH-2 cytokine supposed to limit HIV-1 replication *in vivo *through inhibiting the secretion of inflammatory cytokines (IL-1, TNF-alpha, IL-6, IL-8, IL-12) by lymphoid cells [10]. IL-10 receptor transcripts (IL-10Ra, IL-10Rb) have not been detected in Tat-treated epithelial cells. IL-19 and IL-20 are cytokines identified as IL-10 homologs that share the same receptor complex, indicating that the biological activities of the two cytokines overlap. Both appear to regulate development and proper functioning of the skin. Because the simultaneous presence of both receptor subunits (IL-20R1 and IL-20R2) is requiredfor IL-19 activity in a cell, only tissues that express bothsubunits represent potential targetsfor IL-19. The skin, testis, ovary, heart, lung, muscle, placenta, adrenal gland, small intestine, and salivary gland appear to express both receptors [50]. Expression of both IL-19 receptorsubunits in skin is of a particular interest since IL-19 is up-regulated in psoriatic skin. As for IL-10, the pattern of expression of IL-19 and IL-20 indicates that these cytokines may be involved in the regulationof inflammatory responses. Up-regulation of IL-10 has been associated with the mother-to-child transmission of human cytomegalovirus [51], with peak replication of feline immunodeficiency virus in vivo [52], and in HIV-infected patients [53]. Up-regulation of IL-19 mRNA has been observed in Epstein-Barr virus-transformed lymphocytes [54].

## Conclusion

Extracellular Tat has a mitogenic effect on mammary and amniotic epithelial cells and stimulates the expression of genes of pathogenetic significance in HIV infection. Of particular interest is that Tat enhances the expression of anti-inflammatory cytokines of the IL-10 family. These multiple activities may also help explain the mother-to-child transmission of HIV.

## Methods

### Cell cultures

Four lines of mammary epithelial cells have been used: the spontaneously immortalized MCF-10A cells (non-tumorigenic), the carcinoma-derived MCF-7 (tumorigenic, hormone-dependent) cells, the MEC-1 (SV40-immortalized, non-tumorigenic) and MEC-2 (SV40-immortalized, tumorigenic, hormone-independent) cells. MEC-1 and MEC-2 cell lines have been obtained at our laboratory (32). In addition, the AV-3 line of amniotic origin was used. Cells were cultured in a 1:1 mixture of Dulbecco's modified Eagle's medium and F12 medium (DMEM/F12) supplemented with 10% heat-inactivated fetal bovine serum (FBS; HyClone Laboratories, Logan, UT), 4 mM L-glutamine, 1 mM Na-pyruvate, penicillin and gentamicin. Cell cultures were incubated at 37°C in a humidified atmosphere containing 5% CO_2_.

Unless otherwise specified, tissue culture reagents and chemicals were from Sigma Chemical Co. (St. Louis, MO). Plasticware was obtained from Falcon (Oxnard, CA). Molecular biology reagents were from Applied Biosystems (Monza, Italy).

### Tat treatment of cells grown in low-serum-medium (LSM)

Purified recombinant HIV-1 two-exon Tat produced in *E. coli *(aa 1-101) was obtained from Intracel (London, UK). Synthetic Tat (aa 1–86) was chemically synthesized following established procedures (4). Tat was dissolved at 10 μg/ml in DMEM/F12 containing 1 mM dithio-L-threitol. Tat preparations were shown to be free of endotoxin contamination by the limulus amebocyte lysate assay (BioWhittaker Inc., Walkersville, MD). Aliquots were frozen at -70°C. The two preparations gave equivalent results and were equally neutralized both by a monoclonal antibody (1 μg/ml clone tMab-B, IgG1; Intracel) and by rabbit anti-Tat serum (Intracel).

For studying the effects of Tat on cell growth and gene expression, cells were plated in complete medium for 24 h and – before Tat treatment – incubated overnight in LSM to limit the activity of serum growth factors. LSM consisted of DMEM/F12 containing 0.5% SITE-3 supplement (Sigma), 0.3% dialyzed FBS (10,000 M.W. cut-off), 0.3% human serum albumin, human transferrin (5 μg/ml), Na-selenite (5 ng/ml). Cell proliferation and gene expression profiles were compared in control cultures devoid of Tat and in cultures treated with different concentrations of the Tat protein (10–500 ng/ml). For cell proliferation assays, cultures were exposed to Tat for 1 to 4 days. For analyzing gene expression profiles, cultures were exposed to Tat for 1 to 12 hr.

### Cell proliferation assays

#### A) Cell counts

Five to 7 × 10^4 ^epithelial cells were plated for 24 hrs in wells of 24-well plates (1.9 square cm) with 0.5 ml of DMEM/F12 containing 10% FCS. Cultures were then incubated overnight in LSM. The medium was substituted with LSM containing either no Tat or increasing Tat concentrations (10–500 ng/ml). Cultures were examined 24, 48, 72, and 96 h after treatment with an inverted microscope equipped with a 37°C incubation chamber. Images were acquired with a 10× objective from 6–8 random fields per culture and recorded with a digital camera. Cell counts were obtained with a computerized image analysis system (Image DB; Amplimedical, Mira, Italy) and expressed as number of cells/well. Each bar represents the average of three different tests. In Figure 3, standard deviations of the mean are not reported, but were within 12% of mean values.

In some experiments, the growth-stimulating activity of Tat was neutralized with a monoclonal antibody to Tat (clone tMAb-B, 1 μg/ml) or with Tat rabbit antiserum (1:300 – 1:600 dilution). As a specificity control, a non-relevant neutralizing mAb to coxsackievirus B4 (clone 356.1, 3 μg/ml) and rabbit neutralizing serum to coxsackievirus B4 (1:300) were used. Control antibodies did not influence the mitogenic activity of Tat.

#### B) XTT reduction assay

The assay is based on the ability of viable cells to cleave the tetrazolium ring of the XTT sodium salt (Sigma) generating a water-soluble orange compound. Briefly, 1–3 × 10^4 ^epithelial cells per well were cultured for 24 hrs in flat-bottom 96-well microtiter plates using DMEM/F12 containing 10% FCS. Cultures were then incubated overnight in LSM to free cells from growth factors present in complete medium. The medium was then substituted with LSM without phenol red containing either no Tat or increasing Tat concentrations (10–500 ng/ml). When stimulated cultures were sub-confluent (1–3 days after treatment), XTT (200 μg/ml) was added to each well and cultures were further incubated for 3 hrs. Absorption values were measured at 450 nm with an ELISA reader. Tests were done in quadruplicate. As reported for cell count assays, the Tat neutralizing activity of tMAb-B and Tat rabbit antisera was also evaluated by the XTT assay.

### Analysis of gene expression by RT-PCR

Semiquantitative RT-PCR was used to evaluate the expression of selected mRNA transcripts in cell cultures grown with LSM either in the absence or in the presence of Tat (100 ng/ml). Cultures in T-25 flasks were treated with Tat for different times (1, 3, 6, 12 h). Total RNA was extracted from 3–6 × 10^6 ^cells by the guanidinium thiocyanate method (Life Technologies, Gaithersburg, MD). Experiments were run in duplicate. Total RNA was treated for 1 h at 37°C with DNAse I 2 U/ml in a buffer containing RNAse inhibitor and MgCl_2 _2 mM. DNAse I was inactivated for 5 min at 90°C. cDNA was obtained from 2 μg of RNA with Mo-MLV reverse transcriptase in conjunction with random hexamer primers (Clontech, Palo Alto, CA). Cytokine-specific primer pairs obtained by Clontech (Cytokine MAPPing Amplimers) were used to amplify a variety of cytokines. Reagents from Maxim Biotech (San Francisco, CA) were used to detect transcripts of vascular endothelial growth factor (VEGF 121, 165, and 189 isoforms) and the VEGF receptor-2 (Flk/KDR). A series of published primers specific to HIV receptors, growth-factors, and a factor involved in pre-mRNA maturation were used (Table 1). Expression of the following transcripts was analyzed: IL-6, IL-6-R, IL-8, TGF-beta1, TGF-beta2, CD4, CXCR4, CCR1, CCR2, CCR3, CCR4, CCR5, CPSF3 (cleavage and polyadenylation specificity factor-3). The Applied Biosystems model 2400 thermal cycler was used for PCR reactions using AmpliTaq Gold polymerase (2 units) in a final volume of 50 μl. Samples were denatured at 94°C for 10 minutes before cycling for 21–25 cycles. Amplicons were analyzed on 2% agarose gel using ethidium bromide staining and were photographed on a transilluminator (Kodak Image Station440). Amplicons were quantified using the Kodak 1D 3.5 software using beta-actin and GAPDH transcripts to normalize the data. For selected genes (IL-6, IL-6-R, TGF-beta1, VEGF isoform 165), the relative gene expression obtained by microarray technology was confirmed using real-time PCR. A real-time PCR instrument (Smart Cycler; Cepheid, Sunnyvale, CA) was used together with SYBR Green PCR Master Mix and AmpliTaq Gold DNA polymerase obtained from Applied Biosystems. Reactions were carried out in a final volume of 25 μl. No-amplification control tubes containing samples, but no enzyme were included in each run to exclude the presence of fluorescent contaminants. Conditions consisted of an initial 10-min hold at 95°C followed by 40 amplification cycles. Real-time data were collected during the extension step of each cycle. Amplification of human beta-actin was used as a positive control and for normalizing data. Upon normalization with Ct values of beta-actin amplification, threshold cycle numbers (Ct) obtained for Tat-treated cultures were compared to those obtained for untreated control cultures.

### Analysis of gene expression by gene arrays

GE Array™ Q series membranes (HS-001, HS-003, and HS-015 non-radioactive kits; Superarray, Bethesda, MD) were used to characterize gene expression profiles associated with Tat treatment. Membranes contained 96 cDNA tetraspots of human genes associated with specific biological pathways (cell cycle, common cytokines, inflammatory cytokines and receptors). pUC18 DNA was used as a negative control, and housekeeping genes (beta-actin, cyclophillin A, ribosomal protein L13a, GAPDH) as positive controls. To evaluate the pattern of induced or suppressed gene expression in response to Tat treatment, side-by-side hybridizations with samples from untreated and Tat-treated cultures were performed. Total RNA was extracted from confluent cell monolayers grown with LSM in T-25 flasks and treated or not with Tat. Preliminary experiments with Tat treatment given from 1 to 12 hr, indicated that 4 h was the optimal treatment time. Biotinylated probe synthesis was performed using mixtures of primers proper to each gene set. Before probe synthesis, RNA samples were analyzed and quantified by agarose gel electrophoresis. RNA samples (1–5 μg) combined with the primer mix were added with a prewarmed labeling mix containing 50 units of Mo-MLV reverse transcriptase, 20 units of RNase inhibitor and a dNTP mix containing 2 nmol of biotin-16-dUTP (Roche Applied Science, Monza, Italy) and incubated at 42°C for 90 min. GE Array membranes were prehybridized for 1 h with GEAhyb buffer containing 100 μg/ml heat-denatured salmon sperm DNA (Life Technologies) to prevent non-specific hybridization. Membranes were hybridized overnight at 60°C with denatured cDNA probes using the kit hybridization buffer and hybridization bottles rotating at 10 rpm. After extensive washing (60°C) at low and high stringency conditions in rotating hybridization bottles (10 rpm), membranes were incubated for 10 min with alkaline phosphatase-conjugated streptavidin (AP-streptavidin 1:7.500). Gene expression was detected by chemiluminescence using the AP substrate CDP-Star. Chemiluminescence signals were recorded on X-OMAT film (Kodak, Rochester, NY) using exposure times of 0.5 to 8 minutes. After development, X-OMAT films were scanned with a high-definition scanner (Coolscan 4000 ED, Nikon, Tokyo, Japan). Dedicated software (Gene-Analyzer, Superarray Inc.) was used for densitometric analysis. Experiments on MCF-7 and AV-3 cells were repeatedthree times.

## Authors' contributions

AAB carried out tissue culture work, molecular methods, performed statistical analysis, drafted the manuscript giving critical contributions to the analysis of data and literature.

AB carried out tissue culture work, molecular methods, performed statistical analysis drafted the manuscript giving critical contributions to the analysis of data and literature.

RSA carried out immunological experiments, gave critical contributions to the analysis of data.

FB carried out tissue culture experiments, designed molecular reagents, gave critical contributions to the analysis of data.

AQT conceived the study, set up the methods, helped perform experiments, supervised data acquisition, and prepared the manuscript.

## Figures and Tables

**Table 1:** Primers used to evaluate the expression of mRNA transcripts by RT-PCR.

Gene	GenBank Accession No.		Primer 5'-3' sequence
beta-actin	**NM001101**	Fwd	ATCTGGCACCACACCTTCTACAATGAGCTGCG
		Rvs	CGTCATACTCCTGCTTGCTGATCCACATCTGC
CD4	**BC025782**	Fwd	GTGAACCTGGTGGTGATGAGAGC
		Rvs	GGGGCTACATGTCTTCTGAAACCGGTG
CXCR-4	**BC020968**	Fwd	CTGAGAAGCATGACGGACAAGTACAG
		Rvs	CAACAGCTTCCTTGGCCTCTGACT
CCR-1	**NM001295**	Fwd	GGAAACTCCAAACACCACAGAGGA
		Rvs	AAGATCTCGCTGTACAAGCCTGTG
CCR-2	**NM000647**	Fwd	CTCTCCCATTGTGGGCTCACTCTG
		Rvs	GCAAACACAGCATGGACAATAGCC
CCR-3	**AF026535**	Fwd	CTATGATGACGTGGGCCTGCTC
		Rvs	AAGATCTCGCTGTACAAGCCTGTG
CCR-4	**NM005508**	Fwd	CACCAAAGAAGGCATCAAGGCAT
		Rvs	TGCCACTGTAAAAGCCCACCAAG
CCR-5	**NM000579**	Fwd	CCTGATAAACTGCAAAAGGCTGAAG
		Rvs	AGCAAACACAGCATGGACGACAG
IL-6	**NM000600**	Fwd	ATGAACTCCTTCTCCACAAGCGC
		Rvs	GAAGAGCCCTCAGGCTGGACTG
IL-6R	**X12830**	Fwd	CATTGCCATTGTTCTGAGGTTC
		Rvs	AGTAGTCTGTATTGCTGATGTC
IL-8	**NM000584**	Fwd	ATGACTTCCAAGCTGGCCGTGGCT
		Rvs	TCTCAGCCCTCTTCAAAAACTTCTC
TGF-beta 1	**NM000660**	Fwd	GCCCTGGACACCAACTATTGCT
		Rvs	GGACGGGGATGTAAACCTCGGA
TGF-beta 2	**M19154**	Fwd	GATTTCCATCTACAAGACCACGAGGGACTTGC
		Rvs	GCTTACCGAGAGGAAGCTACATTGACTACGAC
CPFS3	**AF171877**	Fwd	AATGGCTGGCAAACCCTTCTAATG
		Rvs	CATCGTCTTCACTTCCCTCTTCACA

**Table 2:** Expression of HIV receptors and co-receptors in mammary cells exposed to exogenous Tat protein (100 ng/ml)^1^.

**Cell line**	**CD4**	**CXCR4**	**CCR1**	**CCR2**	**CCR3**	**CCR4**	**CCR5**
							
MCF-7 (untreated)	+	+	-	-	-/+	-/+	-
MCF-7 + Tat							
1 h	+	++	-	-	+	+	-
3 h	+	++	-	-	++	++	-
6 h	+	++	-	-	++	++	-
							
MEC-1 (untreated)	+	+	-	-	-/+	-/+	-
MEC-1 + Tat							
1 h	+	++	-	-	+	+	-
3 h	+	++	-	-	++	++	-
6 h	+	++	-	-	++	+	-

1. Expression of mRNA transcripts has been evaluated by semi-quantitative RT-PCR as reported in the Materials and Methods section. Transcript expression is reported as negative (-) or positive. Band intensity is indicated with 1+, 2+, or 3+.

**Table 3:** Expression profile of cell cycle-related genes in epithelial cell lines exposed for 4 h to exogenous Tat protein (100 ng/ml).

**Gene name**	**GenBank accession No.**	**Description**	**Fold change in expression^1^**		
			MCF-7 cell line mean ± SD	AV-3 cell line mean ± SD	Mean of the two cell lines

P21Waf1 (p21Cip1)	**L47233**	Cyclin-dependent kinase inhibitor 1A (p21, Cip1)	4.2 ± .8	5.0 ± .8	4.6
Cdk7	**NM 001799**	Cyclin-dependent kinase 7	3.8 ± .9	5.2 ± .7	4.5
Cul1	**NM 003592**	Cullin 1	2.7 ± .6	5.3 ± 1.1	4.0
Cullin-Cul3	**NM 003590**	Cullin 3	2.6 ± .7	5.3 ± 1.3	3.9
CDC37	**U63131**	Cell division cycle 37 (S. cerevisiae) homolog	1.9 ± .3	5.9 ± .9	3.9
cyclinD1	**M64349**	Cyclin D1 (PRAD1 parathyroid adenomatosis 1)	1.8 ± .3	5.8 ± .7	3.8
Cks1p9	**NM 001826**	CDC28 protein kinase 1	2.4 ± .5	4.9 ± 1.2	3.7
Cullin-Cul4A	**AF077188**	Cullin 4A	2.2 ± .4	4.8 ± 1.1	3.5
Cks2	**NM 001827**	CDC28 protein kinase 2	1.6 ± .5	5.2 ± 1.1	3.4
CDC45-like1	**NM 003504**	Cell division cycle 45-like (S. cerevisiae)	5.1 ± 1.4	1.6 ± .4	3.4
CyclinH	**U11791**	Cyclin H	4.0 ± .6	2.6 ± 1.0	3.3
p27Kip1	**U10906**	Cyclin-dependent kinase inhibitor 1B (p27, Kip1)	1.8 ± .2	4.8 ± 1.0	3.3
Cdk8	**X85753**	Cyclin-dependent kinase 8	1.6 ± .3	4.8 ± .6	3.2
CDC34	**L22005**	Ubiquitin-conjugating enzyme, cell division cycle 34	3.0 ± .5	3.3 ± .6	3.1
Chk1	**AF016582**	CHK1 (checkpoint, S. pombe) homolog	1.6 ± .3	4.7 ± .9	3.1
Cyclin G	**X77794**	Cyclin G1	3.2 ± .5	3.0 ± .3	3.1
CyclinG2	**L49506**	Cyclin G2	2.7 ± .6	3.3 ± .5	3.0
Cdc27	**NM 001256**	Cell division cycle 27	2.7 ± .8	3.2 ± .7	3.0
p18	**U17074**	p18 (cdk4 inhibitor) cyclin-dependent kinase inhibitor 2C (p18, inhibits CDK4)	2.3 ± .5	3.6 ± 1.0	3.0
E2F	**U47677**	Transcription factor 1	3.8 ± .9	1.9 ± .6	2.9
RAD17	**NM 002873**	RAD17 (S. pombe) homolog	2.3 ± 1.0	3.3 ± .8	2.8
RAD50	**U63139**	RAD50 (S. cerevisiae) homolog	2.4 ± .4	3.1 ± .8	2.8
Cullin-Cul2	**U83410**	Cullin 2	1.5 ± .5	3.8 ± .8	2.7
E2F-6	**AF059292**	E2F transcription factor 6	2.9 ± .6	2.3 ± .8	2.6
cyclinB2	**NM 004701**	Cyclin B2	1.8 ± .8	3.2 ± .5	2.5
CyclinF	**U17105**	Cyclin F	3.5 ± .8	1.5 ± .6	2.5
PRC1	**NM 003981**	Protein regulator of cytokinesis1	2.0 ± .4	3.0 ± .5	2.5
Cullin-Cul4B	**AB014595**	Cullin 4B	2.0 ± .3	2.9 ± .3	2.5
cyclinE1	**M73812**	Cyclin E1	2.0 ± .4	2.9 ± .3	2.5
Hus1	**NM 004507**	HUS1 (S. pombe) checkpoint homolog	1.8 ± .8	3.1 ± .2	2.5
MPP2	**U74613**	Human putative M phase phosphoprotein 2 (MPP2) mRNA	1.8 ± .6	3.1 ± .6	2.5
Ki67 (MKI67)	**X65550**	Antigen identified by monoclonal Ab Ki-67	1.5 ± .3	3.3 ± .4	2.4
Skp1	**U33760**	Cyclin A / CDK2-associated p19 (Skp1)	1.5 ± .3	3.2 ± .6	2.4
Skp2	**U33761**	Human cyclin A / CDK2-associated p45 (Skp2)	2.0 ± .3	2.7 ± .6	2.4
PCNA	**J04718**	Proliferating cell nuclear antigen	1.9 ± .5	2.8 ± .5	2.4
Bax	**L22474**	Bcl2-associated X protein	1.6 ± .5	3.0 ± .7	2.3
cyclinE2	**NM 004702**	Cyclin E2	1.5 ± .4	3.1 ± .3	2.3
CyclinB	**M25753**	Cyclin B1	1.7 ± .3	3.1 ± .5	2.4
CyclinC	**M74091**	G1/S-specific Cyclin C	1.6 ± .4	3.0 ± .6	2.3
MCM5 (CDC46)	**NM 006739**	Minichromosome maintenance deficient (S. cerevisiae) 5 (cell division cycle 46)	1.8 ± .2	2.7 ± .4	2.3
MRE11A	**U37359**	Meiotic recombination (S. cerevisiae) 11 homolog A	1.5 ± .7	2.9 ± .5	2.2
Cdk6	**X66365**	Cyclin-dependent kinase 6	2.0 ± .3	2.3 ± .9	2.2
Nibrin	**AF058696**	Nijmegen breakage syndrome 1 (nibrin)	1.8 ± .4	2.5 ± .5	2.2
Rbx1	**NM 014248**	Homo sapiens ring-box protein 1 (RBX1) mRNA	1.5 ± .7	2.8 ± .6	2.2
MCM2	**D83987**	Minichromosome maintenance deficient (S. cerevisiae) 2 (mitotin)	1.5 ± .3	2.7 ± .3	2.1
MCM4 (CDC21)	**X74794**	Minichromosome maintenance deficient (S. cerevisiae) 4	2.0 ± .4	1.8 ± .2	1.9
MCM7 (cdc47)	**D28480**	Minichromosome maintenance deficient (S. cerevisiae) 7	1.5 ± .5	2.3 ± .7	1.9
c-abl	**X16416**	V-abl Abelson murine leukemia viral oncogene homolog 1	1.6 ± .5	1.9 ± .6	1.7
Mdm2	**Z12020**	Mouse double minute 2, human homolog of p53-binding protein	1.6 ± .6	1.8 ± .6	1.7
Rpa	**L07493**	Replication protein A3 (14 Kd)	1.6 ± .5	1.8 ± .4	1.7

1. Mean densitometric values are expressed for each gene as fold difference in cultures exposed to Tat / control cultures not exposed to Tat. Negative (pUC18) and positive controls (GAPDH, cyclophillin A, RPL13A, beta-actin) were used to normalize the data. Data are reported only for genes that were up-regulated by Tat at least 1.5-fold in both cell lines.

**Table 4:** Expression profile of cytokine-related genes in epithelial cell lines exposed for 4 h to exogenous Tat protein (100 ng/ml).

**Gene name**	**GenBank accession No.**	**Description**	**Fold change in expression^1^**		
			MCF-7 cell line mean ± SD	AV-3 cell line mean ± SD	Mean of the two cell lines

MIF	**NM 002415**	Macrophage migration inhibitory factor (glycosylation-inhibiting factor)	5.2 ± .9	2.1 ± 1.8	3.7
IL-19	**NM 013371**	Interleukin 19	4.0 ± 1.1	2.4 ± .3	3.2
IL-13RA1	**NM 001560**	Interleukin 13 receptor. alpha 1	4.1 ± 1.2	2.1 ± .6	3.1
GP130	**NM 002184**	IL-6 signal transducer (gp 130, oncostatin M receptor)	3.9 ± 1.4	2.0 ± .3	3.0
CCR3	**NM 001837**	Chemokine (CC motif) receptor 3	4.2 ± 1.1	1.6 ± .3	2.9
IL-11	**M57765**	Interleukin 11	3.9 ± .7	1.7 ± .4	2.8
SCYE1	**NM 004757**	Small inducible cytokine subfamily E. member 1 (endothelial monocyte-activating)	2.6 ± .5	3.0 ± .6	2.8
BMP2	**NM 001200**	Bone morphogenetic protein 2	3.4 ± .5	2.0 ± .5	2.7
IL-18	**NM 001562**	Interleukin 18 (interferon-gamma-inducing factor)	2.2 ± .6	3.1 ± .9	2.7
TGFb1	**X02812**	Transforming growth factor beta 1 (Camurati-Engelmann disease)	2.8 ± .5	2.1 ± .4	2.5
SCYC1	**U23772**	Chemokine (C motif) ligand 1	2.5 ± .6	1.8 ± .4	2.2
BMP1	**NM 006128**	Bone morphogenetic protein 1	2.7 ± 1.0	1.5 ± .5	2.1
VEGF-C	**X94216**	Vascular endothelial growth factor C	2.5 ± 1.1	1.6 ± .2	2.0
IL-15	**AF031167**	Interleukin 15	2.0 ± .5	2.0 ± .3	2.0
TNFR1	**M33294**	Tumor necrosis factor receptor superfamily. member 1A	2.3 ± .7	1.7 ± .5	2.0
VEGF-B	**U48801**	Vascular endothelial growth factor B	2.2 ± .5	1.8 ± .1	2.0
CXCR4	**NM 003467**	Chemokine (CXC motif) receptor 4	2.1 ± .5	1.5 ± .5	1.8
IL-6	**M14584**	Interleukin 6 (interferon, beta 2)	2.0 ± .6	1.6 ± .2	1.8
IL-10	**M57627**	Interleukin 10 (high-level expression in both cell lines)	1.9 ± .5	1.7 ± .4	1.8
LT-b	**NM 002341**	Lymphotoxin beta (TNF superfamily, member 3)	1.8 ± .6	1.6 ± .8	1.7
PDGFa	**X06374**	Platelet-derived growth factor alpha polypeptide	1.7 ± .7	1.7 ± .5	1.7
LTbR	**L04270**	Lymphotoxin beta receptor (TNFR superfamily, member 3)	1.7 ± .2	1.6 ± .4	1.7
SCYA5 / RANTES	**NM 002985**	Chemokine (CC motif) ligand 5	1.6 ± .3	1.7 ± .5	1.6
IL-20	**NM 018724**	Interleukin 20	1.6 ± .7	1.6 ± .5	1.6

1. Mean densitometric values are expressed for each gene as fold difference in cultures exposed to Tat / control cultures not exposed to Tat. Negative (pUC18) and positive controls (GAPDH, cyclophillin A, RPL13A, beta-actin) were used to normalize the data. Data are reported only for genes that were up-regulated by Tat at least 1.5-fold in both cell lines.
